# A Risk Model Based on Immune-Related Genes Predicts Prognosis and Characterizes the Immune Landscape in Esophageal Cancer

**DOI:** 10.3389/pore.2022.1610030

**Published:** 2022-03-14

**Authors:** Yan Xie, Ruimin Fu, Zheng Xiao, Gang Li

**Affiliations:** ^1^ Department of Clinical Laboratory, Henan Provincial People’s Hospital, Zhengzhou University People’s Hospital, Henan University People’s Hospital, Zhengzhou, China; ^2^ College of Health Management, Henan Finance University, Zhengzhou, China

**Keywords:** immunotherapy, chemotherapy, prognosis, esophageal cancer, immune-related genes, risk model

## Abstract

Aberrant immune gene expression has been shown to have close correlations with the occurrence and progression of esophageal cancer (EC). We aimed to generate a prognostic signature based on immune-related genes (IRGs) capable of predicting prognosis, immune checkpoint gene (ICG) expressions, and half-inhibitory concentration (IC_50_) for chemotherapy agents for EC patients. Transcriptome, clinical, and mutation data on tumorous and paratumorous tissues from EC patients were collected from The Cancer Genome Atlas (TCGA) database. Then, we performed differential analysis to identify IRGs differentially expressed in EC. Their biofunctions and related pathways were explored using Gene Ontology (GO) and Kyoto Encyclopedia of Genes and Genomes (KEGG) enrichment analyses. These gene expression profiling data were merged with survival information and subjected to univariate Cox regression to select prognostic genes, which were then included in a Lasso-Cox model for signature generation (risk score calculation). Patients were divided into the high- and low-risk groups using the median risk score as a cutoff. The accuracy of the signature in overall survival prediction was assessed, so were its performances in predicting ICG expressions and IC_50_ for chemotherapy and targeted therapy agents and immune cell landscape characterization. Fifteen prognostic IRGs were identified, seven of which were optimal for risk score calculation. As expected, high-risk patients had worse overall survival than low-risk individuals. Significant differences were found in tumor staging, immune cell infiltration degree, frequency of tumor mutations, tumor mutation burden (TMB), and immune checkpoint gene expressions between high- vs. low-risk patients. Further, high-risk patients exhibited high predicted IC_50_ for paclitaxel, cisplatin, doxorubicin, and erlotinib compared to low-risk patients. The seven-IRG-based signature can independently and accurately predict overall survival and tumor progression, characterize the tumor immune microenvironment (TIME) and estimate ICG expressions and IC_50_ for antitumor therapies. It shows the potential of guiding personalized treatment for EC patients.

## Introduction

EC is the seventh most common cancer and the sixth leading cause of death from cancer across the world [1]. Current treatment strategies can vary among stages or cancer types, with endoscopic resection optimal to most early tumors, surgical resection followed by chemotherapy, radiotherapy, or targeted therapy (single or combined) beneficial to locally advanced tumors, but non-surgical treatment, particularly systemic chemotherapy, suitable for metastatic cases [2]. However, the prognosis remains constant in EC patients undergoing antitumor treatment, though diverse and being improved [2]. EC is often diagnosed when it develops to middle or advanced stages and when a 5-year survival rate decreases to lower than 20% [3]. Even so, immunotherapy, particularly immune checkpoint inhibitors (ICIs), has received much attention over the last few years. It has been proven effective in and is recommended for the management of malignant melanoma, non-small cell lung cancer, and clear cell renal cell carcinoma, with significantly improved patient prognosis [4]. For EC treatment, ICIs, alone or combined with chemoradiotherapy, have gradually moved up to the first-line treatment and exerted impressive antitumor effects [5, 6]. However, not all patients can benefit from this treatment, despite some patients who have shown better overall or progression-free survival, which is a concern for immunotherapy application. Therefore, biomarkers for systemic prediction of prognosis and immunotherapy efficacy for EC patients are urgently needed [7].

Tumor occurrence and progression are often determined by malignant behaviors, such as tumor cell proliferation and invasion, which are related to the tumor microenvironment (TME), especially the tumor immune microenvironment (TIME) that contains immune suppressor cells helpful to immune escape [8]. Immune checkpoint molecules, particularly programmed death receptor 1 (PD-1) and programmed cell death-ligand 1 (PD-L1), have been proven to be more frequently present in tumor cells and tumor-infiltrating immune cells to disturb antitumor immune response [9]. Their involvement in cell differentiation in EC cells, tumor staging, immunotherapy efficacy, and patient prognosis have been extensively reported [10-13]. Immune cell infiltration patterns characterized by these molecules can be associated with tumorigenesis, progression, and lymph node metastasis, and subsequently impaired efficacy of immunotherapy and prognosis [14-16]. Further, high TMB is positively correlated with overall survival (OS) after ICIs treatment in various cancer types [17], which may predict the efficacy of ICIs more accurately than PD-L1 [18].

Compared to a single cancer-related gene with limited predictive power, a multigene signature has more significant implications in this regard. In this study, we generated a prognostic risk model comprising seven IRGs based on differentially expressed IRGs in EC, validated its performances in overall survival prediction, and assessed its correlations with prognostic clinicopathological and immune features.

## Materials and Methods

### Data Acquisition

RNA-seq, clinical, and tumor mutation data on 171 tissue samples (160 tumorous and 11 paratumorous tissues) from EC patients were collected from the Cancer Genome Atlas-Genomic Data Commons (TCGA-GDC; https://portal.gdc.cancer.gov/), among which incomplete clinical data were improved by the University of California Santa Cruz (UCSC) the Cancer Genome Atlas (TCGA) browser (https://xenabrowser.net/datapages/). Data from EC patients, including 77esophageal adenocarcinoma (EAC) cases and 76esophageal squamous cell carcinoma (ESCC) cases, were obtained from this database. Log2 transformed expression scores of each gene were averaged, and samples with an invalid expression score of 0 were removed. Standardized RNA transcription expression profiles were obtained. Furthermore, samples with a follow-up of fewer than 30 days or incomplete follow-up records that may affect subsequent analyses were excluded. The list of IRGs for EC was retrieved from the Immunology Database Information Portal (https://www.immport.org/resources). The raw data for all included EC cases are summarized in Supplementary File S1.

### Differential Analysis of Immune-Related Genes

The LIMMA package in the R was utilized to carry out differential analysis for differentially expressed genes (DEGs) in EC vs. paratumorous tissues (log2 fold-change [FC] > 1.0 and false discovery rate [FDR] < 0.05) [19]. The volcano plot for these genes was drawn with GGPLOT2 package in R [20]. DEGs and IRGs were intersected to obtain differentially expressed IRGs (DEIs) and their expression data, which were visualized in the Venn plot and heatmap with VENN and pheatmap R-package [21]. Gene Ontology (GO) and Kyoto Encyclopedia of Genes and Genomes (KEGG) enrichment analysis were performed to identify biofunctions and pathways related to DEIs with clusterProfiler package in R (*p* < 0.05) [22], and the results were plotted with GGPLOT2 R-package [20].

### Establishment and Validation of the Prognostic Signature

All patients (each case contains complete data of RNA-seq and survival information) were randomly categorized into the training (*n* = 104) and test cohorts (*n* = 50) for signature generation and validation, respectively. DEIs expression information with survival data was integrated and imported to univariate Cox regression to screen prognostic genes (*p* < 0.05). These genes were included in a Lasso-Cox model for cross-validation with a random stimulation of 1,000 times. Genes with a low correlation to prognosis were excluded to prevent over-fitting, and the gene group with the smallest error was considered optimal for signature generation. The risk score was calculated with glmnet R-package [23]. The median risk score of the training cohort was used as a cutoff for risk stratification (high- and low-risk groups in the training, test, and combined cohorts). Besides, clinicopathological characteristics were compared between the training and test cohorts to ensure no differences between them (*p* < 0.05). For validation, we compared the overall survival (OS) of high- and low-risk EC patients using Kaplan-Meier (KM) survival analysis and receiver operating characteristic (ROC) curves with survminer and timeROC R-package [24]. The accuracy of the signature was determined by the area under ROC curve (AUC) values. Clinical data (age, gender, tumor differentiation, histological grading and subtype, and TNM staging) with risk scores were merged. Risk curves were plotted with pheatmap R-package to identify high-risk DEIs associated with worse OS. We also performed KM survival analyses of patients with EAC and ESCC pathological subtypes. Furthermore, patients with complete information on clinical characteristics were selected to assess the independence of the risk score in prognosis prediction. Differential analyses of the risk score between subgroups of each clinicopathological parameter (age, gender, tumor differentiation, TNM staging, and histological grading) were performed using Wilcoxon rank-sum test with LIMMA R-package to examine the effect of the signature on tumor progression [19], and the results were visualized with GGPLOT2 R-package [20].

### Tumor Immune Microenvironment and Tumor Mutation Burden Analyses

For TIME characterization, we analyzed the infiltration degree and pattern of 22 immune cell subpopulations with Cell-type Identification By Estimating Relative Subsets Of RNA Transcripts (CIBERSORT) [25]. Transcriptomic expression scores were converted into immune cell infiltration information (*p* < 0.05) for more specific comparisons of immune cell infiltration differences between high-vs. low-risk groups, and the results were visualized with GGPLOT2 R-package [20]. EC mutation data were analyzed to yield TMB and the frequency of gene mutations for subsequent analyses. The former was combined with survival data of high- and low-risk patients for difference analysis with GGPLOT2 R-package [20]. The waterfall plot was generated with Maftools R-package to compare differences in gene mutation frequency between high- vs. low-risk patients [26]. Survival differences between high vs. low TMB were compared using KM survival analysis with R-package survivminer package in R.

### Immune Checkpoint Gene Expressions and IC_50_ for Chemotherapy Agents

Immune checkpoint molecules, including inhibitory and stimulatory immune checkpoint molecules, are defined as ligand-receptor pairs that exert inhibitory or stimulatory effects on immune responses. We compared expressions of the 12 most common immune checkpoint genes (e.g., *CD200*, *CD200R*, *CD274 [encoding PD-L1]*, *CD96*, *CTLA4*, *DNAM-1*, *IDO1*, *LAG3*, *NKG2A, PDCD1 [encoding PD1]*, *TIGIT*, and *VISTA*) between the high-vs. low-risk groups with R-package LIMMA [19]. Gene expressions were visualized with R-package GGPLOT2 [20]. Then, we predicted IC_50_ for three first-line chemotherapy agents (paclitaxel, cisplatin, and doxorubicin) and the most commonly used targeted therapy agent erlotinib between the two risk groups with LIMMA and pRophetic R-package [19].

### Statistical Analysis

All statistical analyses were carried out in R (version 4.0.2). The utilized R-package including LIMMA, GGPLOT2, clusterProfiler, glmnet, survivminer, timeROC, pheatmap, ggpubr, Maftools, pRophetic, and VENN [19-26]. The Log-rank test was applied for survival analysis, the Wilcoxon rank-sum test was employed for differential analysis. A two-tailed *p*-value of <0.05 was considered statistically significant.

## Results

### Differentially Expressed Immune-Related Genes

**FIGURE 1:**
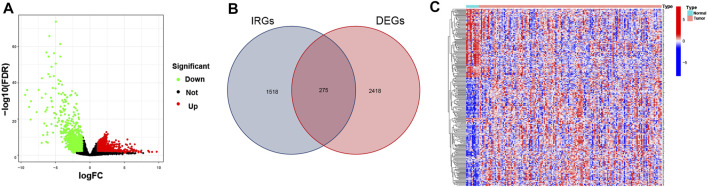
Differentially expressed immune-related genes (IRGs) in EC. **(A)** The Volcano plot shows IRG expressions in EC. Green dots represent down-regulated genes, red dots stand for up-regulated genes, and black dots indicate nondifferential expressed genes (nonDEGs). **(B)** The Venn diagram shows the distribution of DEGs and IRGs in EC. **(C)** The heatmap depicts IRG expressions in EC and paratumorous tissues. Blue blocks suggest low gene expression, and red blocks refer to high gene expression.

**FIGURE 2:**
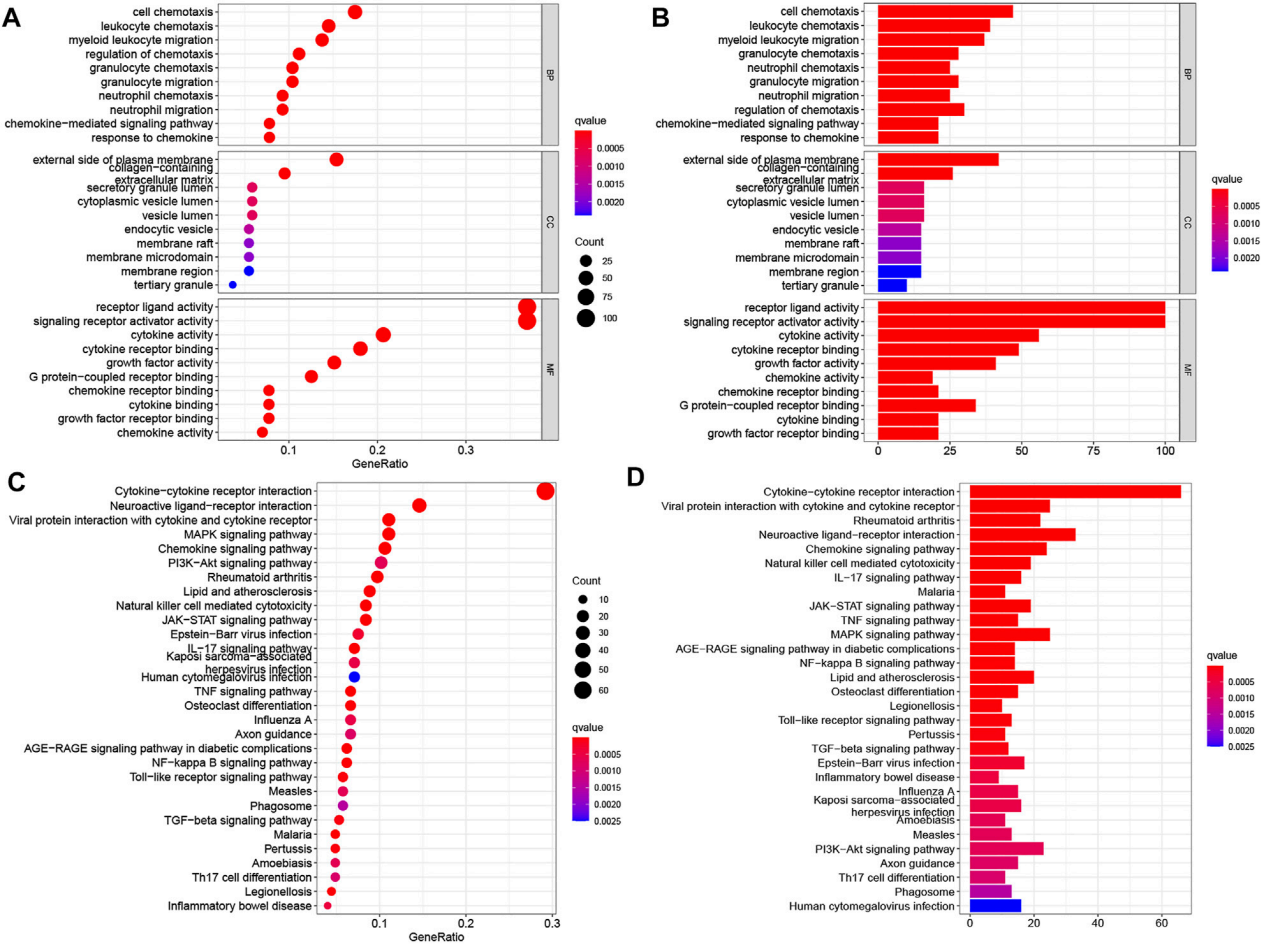
Enrichment analyses of differentially expressed IRGs in EC. **(A,B)** GO terms and **(C,D)** KEGG pathways enriched in differentially expressed IRGs. In panels **(A,C)**, the abscissas of bubble diagrams denote the proportion of genes, and bubble size indicates the number of enriched genes. The color from blue to red represents an increasing significance level. In panels **(B,D)**, the abscissas of the histograms denote the number of genes. The color from blue to red indicates an increasing significance level.

The differential analysis of normalized transcriptomic expression data on 160 EC and 11 paratumorous tissue samples showed 2,693 DEGs associated with EC. Their distribution between EC vs. paratumorous tissues was visualized in the volcano plot (Figure 1A). In the Venn plot, 275 DEIs (Figure 1B) were identified after intersection of DEGs with 1,793 IRGs, including 109 down-regulated genes and 166 up-regulated ones. The thermogram revealed that expression levels of these DEIs were markedly different in EC vs. normal tissues (Figure 1C). As for mechanisms for DEIs in EC, biological process (BP) terms were mostly enriched in leukocyte chemotaxis, migration, regulation of chemotaxis, and chemokine-mediated signaling pathways. The top three terms of cellular components (CC) comprised lateral plasma membrane, extracellular matrix, and secretory granule cavity. The molecular function (MF) of these DEIs mainly focused on receptor-ligand binding activity, receptor signal activator activity, cytokine, growth factor, and chemokine activity (Figures 2A,B). These genes primarily were associated with cytokine-receptor interaction, chemokine signaling pathway, and the MAPK signaling pathway. Therefore, the 275 DEIs were correlated with immune function, as supported by the above GO terms and KEGG pathways (Figures 2C,D).

### Signature Generation

**FIGURE 3:**
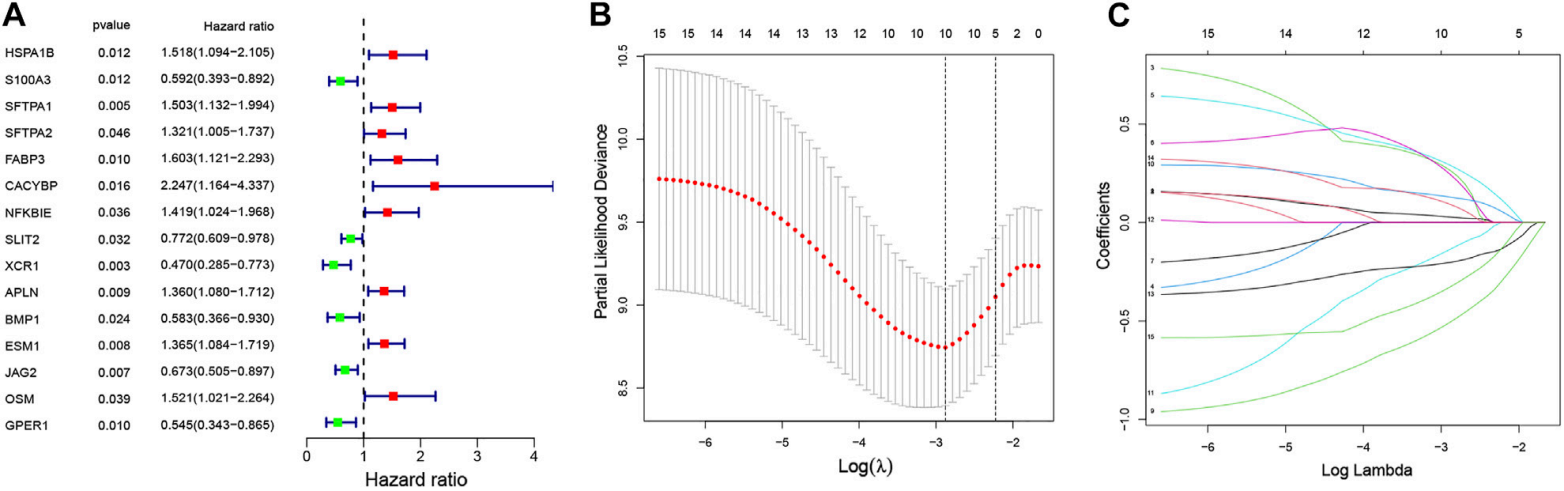
A seven-IRG-based prognostic signature. **(A)** The forest plot reveals 15 prognostic IRGs identified using univariate Cox regression and their correlation pattern with overall survival time. **(B)** Survival cross-validated partial log-likelihood deviance for assessment of the fit of the Cox model. **(C)** Evaluation of the change in risk (HR trajectory) using Lasso regression. The abscissa and ordinate represent log-transformed (or independent) variables and coefficients of independent variables, respectively.

In univariate Cox regression analysis, 15 of 275 IRGs markedly correlated with patient survival were confirmed. The forest plot displayed correlation patterns of the 15 IRGs with survival time (Figure 3A). Ultimately, seven prognostic IRGs (*APLN*, *CACYBP*, *FABP3*, *GPER1, JAG2*, *SFTPA1*, and *XCR1*) were selected by the Lasso-Cox model (Figures 3B,C; Table 1), of which *APLN*, *CACYBP*, *FABP3*, and *SFTPA1* had a hazard ratio (HR) of >1 and were considered high-risk genes of patient overall survival, often associated with a poor prognosis. The remanent three genes (*GPER1*, *JAG2*, and *XCR1*) had an HR of less than 1, indicating that their overexpression was correlated with longer overall survival. Based on expression scores and risk coefficients of the seven hub genes, the risk score of each patient was calculated as follows: rick score = (0.24466 × APLN) + (0.64372 × CACYBP) + (0.52781 × FABP3) + (−0.61487 × GPER1) + (−0.44301 × JAG2) + (0.51644 × SFTPA1) + (−0.76193 × XCR1). Each coefficient numerically represents the hazard weight of gene expression of a differentially expressed gene. In addition, there were no differences in clinicopathological characteristics between the training vs. test cohorts (Table 2), indicating that the gene group can be used for subsequent analysis.

**TABLE 1:** Multivariate Cox regression analysis of seven IRGs associated with overall survival of patients with EC.

Gene ID	Coef	HR	HR 95% low	HR 95% high	*p*-value
SFTPA1	0.51644	1.67605	1.23509	2.27443	0.00091
FABP3	0.52781	1.69521	1.17262	2.45072	0.00500
CACYBP	0.64372	1.90356	0.93207	3.88762	0.07725
XCR1	−0.76193	0.46677	0.26349	0.82686	0.00901
APLN	0.24466	1.27718	0.95850	1.70183	0.09481
JAG2	−0.44301	0.64210	0.46236	0.89171	0.00819
GPER1	−0.61486	0.54071	0.33372	0.87610	0.01252

Coef: regression coefficient; HR: hazard ratio.

**TABLE 2:** Clinicopathological features of EC patients.

Features	Subgroups	Training cohort	Test cohort	Combination cohort	*p*-value
Age	>65 years	39 (37.5%)	19 (38%)	58 (37.66%)	1
	≤65 years	65 (62.5%)	31 (62%)	96 (62.34%)	
Gender	Female	16 (15.38%)	7 (14%)	23 (14.94%)	1
	Male	88 (84.62%)	43 (86%)	131 (85.06%)	
Grade	G1-2	52 (50%)	26 (52%)	78 (50.65%)	1
	G3	28 (26.92%)	14 (28%)	42 (27.27%)	
	Unknown	24 (23.08%)	10 (20%)	34 (22.08%)	
Stage	Stage I–II	54 (51.92%)	27 (54%)	81 (52.6%)	0.7381
	Stage III-IV	39 (37.5%)	16 (32%)	55 (35.71%)	
	Unknown	11 (10.58%)	7 (14%)	18 (11.69%)	
T	T1-2	40 (38.46%)	22 (44%)	62 (40.26%)	0.4876
	T3-4	55 (52.88%)	21 (42%)	76 (49.35%)	
	Unknown	9 (8.65%)	7 (14%)	16 (10.39%)	
M	M0	79 (75.96%)	37 (74%)	116 (75.32%)	0.9899
	M1	6 (5.77%)	2 (4%)	8 (5.19%)	
	Unknown	19 (18.27%)	11 (22%)	30 (19.48%)	
N	N0	42 (40.38%)	19 (38%)	61 (39.61%)	1
	N1-3	52 (50%)	24 (48%)	76 (49.35%)	
	Unknown	10 (9.62%)	7 (14%)	17 (11.04%)	
histology	Adenocarcinoma	51 (49.04%)	26 (52%)	77 (50%)	0.753
	Squamous carcinoma	52 (0.96%)	24 (48%)	76 (49.35%)	
	Serous Neoplasms	1 (50%)	0 (0%)	1 (0.65%)	

T: tumor; M: metastasis; N: node.

### Validation of the Prognostic Signature

**FIGURE 4:**
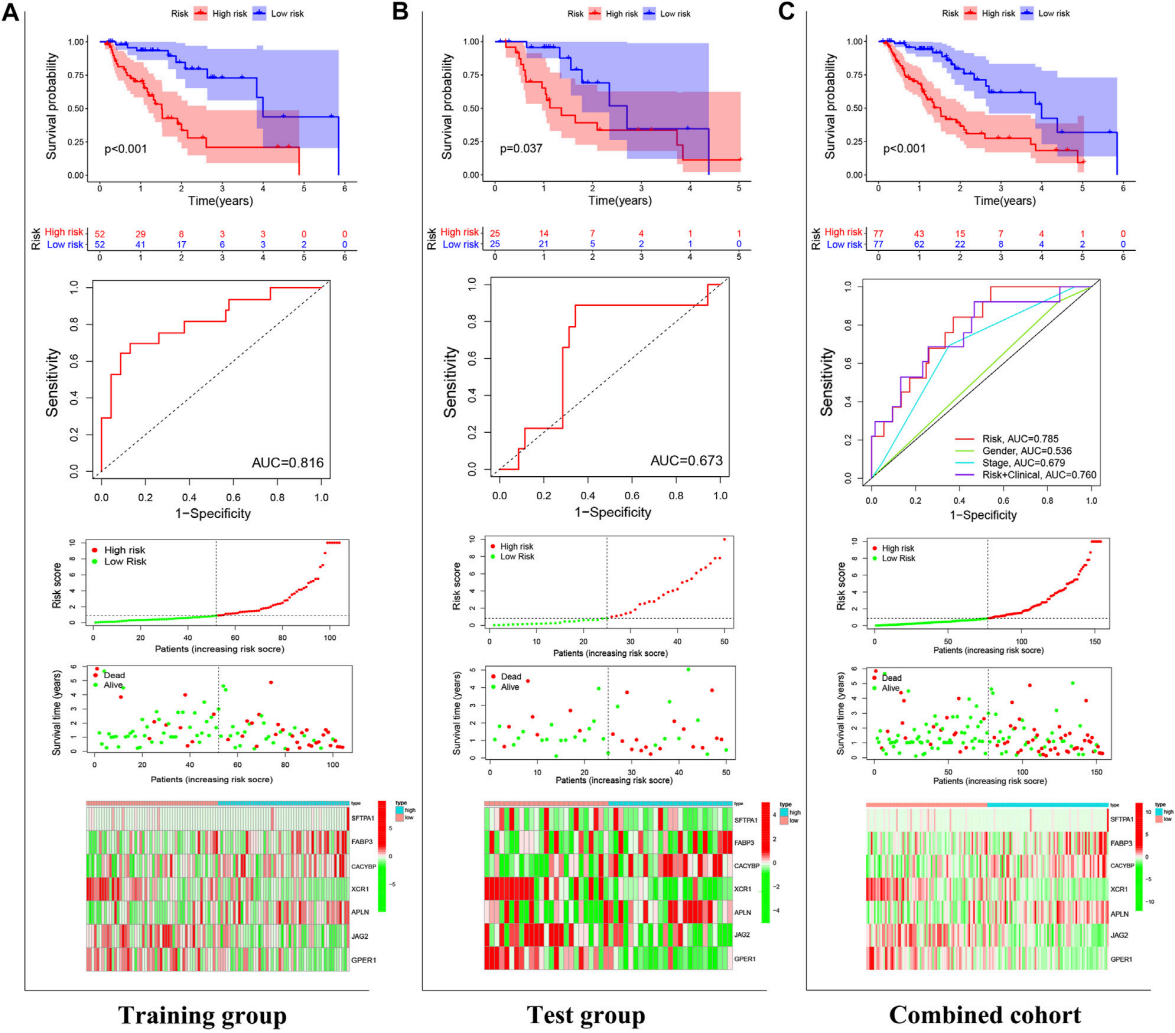
Validation of the prognostic signature. The power of the prognostic signature in predicting the overall survival of patients in **(A)** the test, **(B)** training, and **(C)** combined cohorts using Kaplan-Meier survival analysis, ROC curves, and risk curves. In risk curves, the abscissas represent the number of EC patients, and the ordinates, from top to bottom, indicate gene expressions, risk score, and survival time.

**FIGURE 5:**
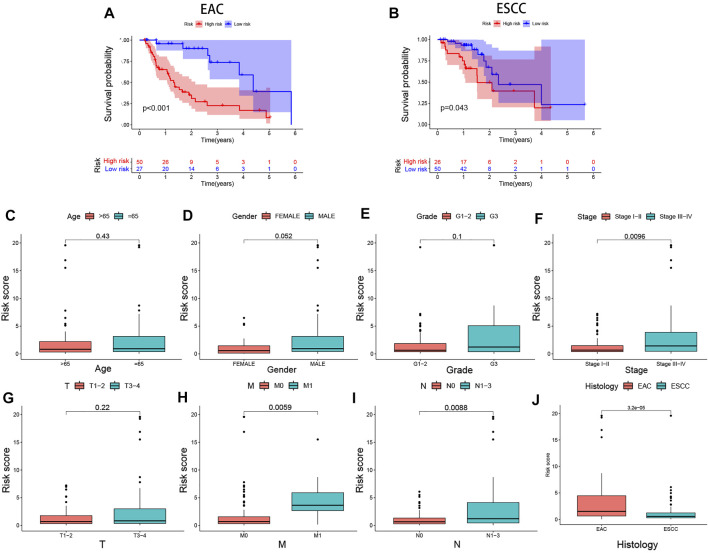
Differential expression analyses of the prognostic signature with clinicopathological characteristics of EC patients. Risk score-based survival analysis in EAC and ESCC cases **(A,B)**. The seven-IRG-based prognostic signature is correlated with **(C)** age, **(D)** gender, **(E)** pathological grading, **(F)** tumor staging, **(G)** tumor size, **(H)** distant metastasis, **(I)** lymph node metastasis, and **(J)** histological subtype. EAC, Esophageal adenocarcinoma; ESCC, Esophageal squamous cell carcinoma.

In the training (*p* < 0.001), test (*p* = 0.037), and combined (*p* < 0.001) cohorts, high-risk patients showed worse overall survival than low-risk patients, with the corresponding AUCs of 0.816, 0.673, and 0.785 (Figures 4A–C), indicating satisfactory sensitivity and specificity of the prognostic signature. Compared to gender (*p* = 0.536) and stage (*p* = 0.679), the risk model revealed greater diagnostic efficiency but did not show better prediction when it was integrated into a clinicopathological-genomic nomogram with gender and stage. High-risk EC patients showed an increased risk score worse overall survival, along with upregulation of four high-risk genes and downregulation of three low-risk genes compared to low-risk patients, comparable in the training and test cohorts (Figures 4A–C). Moreover, subgroup analysis of different pathological subtypes of EC revealed that the OS time of EAC and ESCC patients with high-risk score was shorter than that of patients with low-risk score, and the difference was much more significant in the cases of EAC compared to ESCC (*p*<0.001 vs. *p* = 0.043) (Figures 5A,B). Besides, 120 EC patients had complete clinical information (age, gender, histological grading, histological subtypes, and clinical staging). In univariate Cox regression, gender (*p* = 0.041), clinical staging (*p* < 0.001), and risk score (*p* < 0.001) were prognostic markers in EC, of which gender was excluded in multivariate Cox regression (Table 3). Therefore, clinical staging (*p* < 0.001) and risk score (*p* < 0.0001) could act as independent prognostic factors of EC from other clinical characteristics. All these findings suggested that the seven-IRG-based prognostic signature could accurately predict the prognosis of EC patients.

**TABLE 3:** Univariate and multivariate Cox regression for independence of riskScore and clinicopathological features in prognosis prediction.

Features	Univariate Cox regression				Multivariate Cox regression			
	HR	HR 95% low	HR 95% high	*p*	HR	HR 95% low	HR 95% high	*p*
Age	1.005	0.979	1.031	0.716				
Gender (male vs. female)	7.980	1.094	58.217	0.041	4.382	0.585	32.826	0.150
Grade (I/II vs. III/IV)	1.334	0.813	2.187	0.254				
Stage (I vs. II/III)	2.768	1.779	4.305	<0.001	2.398	1.519	3.786	<0.001
riskScore (high vs.low)	1.167	1.100	1.238	<0.001	1.139	1.072	1.210	<0.001
Histology (EAC vs. ESCC)	0.779	0.407	1.492	0.452				

HR: hazard ratio. EAC: Esophageal adenocarcinoma. ESCC: esophageal squamous cell carcinoma.

### Clinical Implications of the Prognostic Signature

We performed differential analyses of the risk score between clinicopathological subgroups to assess clinical correlations of the signature. The results showed that the risk score increased with EC clinical stage (*p* = 0.0096). High-risk patients had further tumor progression concerning metastasis (*p* = 0.0059) and metastatic lymph nodes (*p* = 0.0088) compared to low-risk patients (Figures 5C–I). Moreover, subgroup analyses showed that EAC patients had a higher risk score than ESCC patients (*p* < 0.001, Figure 5J).

### Mutation Prediction and Immune Cell Landscape Characterization

**FIGURE 6:**
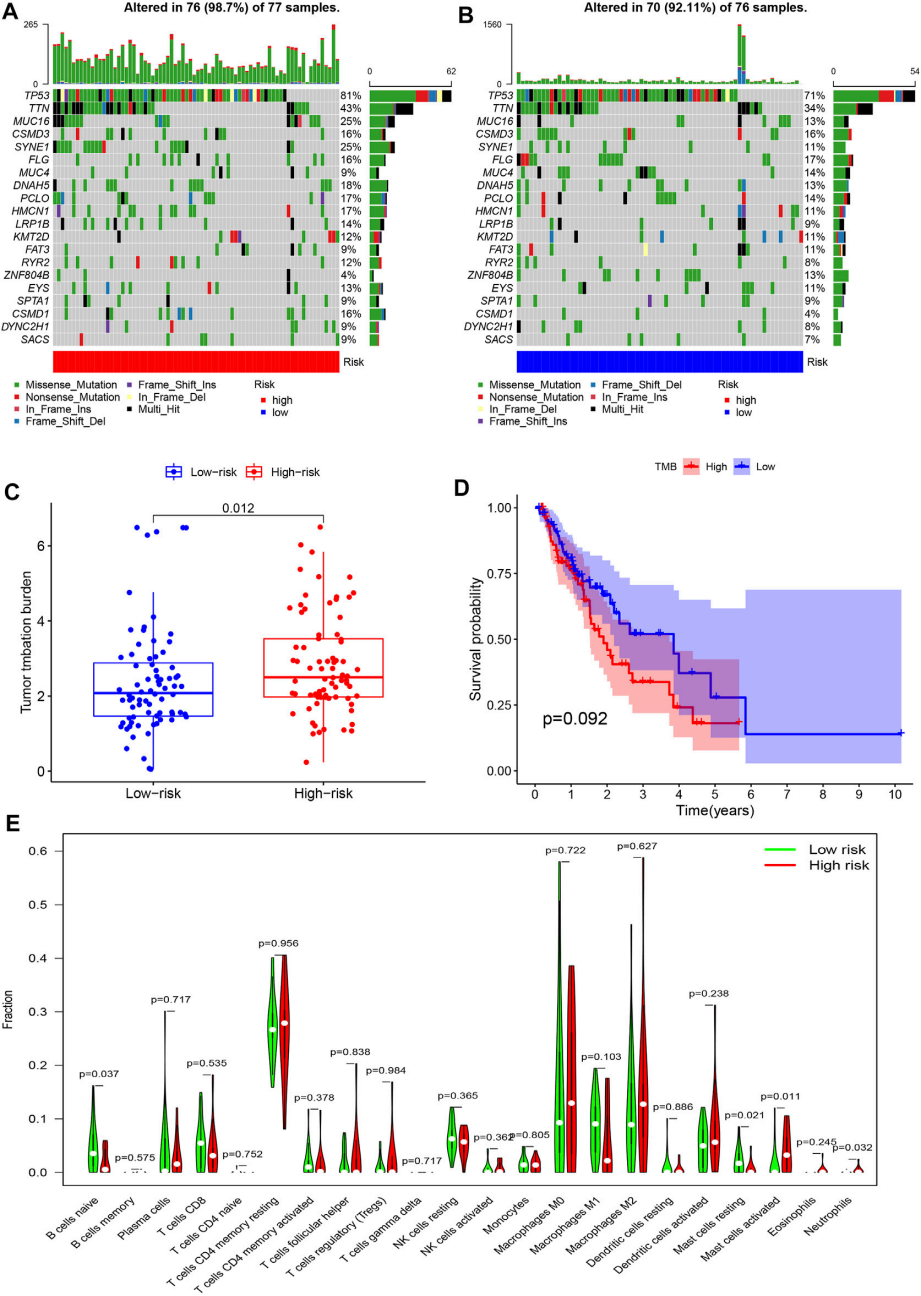
Mutation prediction and immune cell landscape characterization in EC. **(A,B)** Waterfall plots show the frequency of gene mutations in high- and low-risk patients. The abscissas represent the number of patients, and the ordinates denote the name of mutated genes. Different colors of blocks represent different mutation types. **(C)** Differences in TMB between the high- and low-risk groups. **(D)** Kaplan-Meier survival analysis reveals no significant difference in overall survival between high versus low TMB patients. **(E)** Immune infiltration patterns between high- and low-risk patients. The abscissa and the ordinate represent the type and proportion of immune cells.

Most frequently, mutations were identified in *CSMD3*, *MUC16*, *SYNE1*, *TP53*, and *TTN* in both risk groups, which were primarily missense mutations. In intergroup comparisons, high-risk patients had a higher frequency of these mutations except for *CSMD3* (Figures 6A,B) and increased TMB (*p* = 0.012, Figure 6C) compared to low-risk patients. According to the median TMB, all patients were assigned to the high or low TMB group to assess the discrimination ability of TMB in survival prediction. However, no significance in OS was observed between the two groups (*p* = 0.092) (Figure 6D).

As for differences in the TIME, high-risk patients exhibited impaired naive B cell and resting mast cell infiltration compared to low-risk patients. Rather, the degree of activated mast cell and neutrophil infiltration was remarkably enhanced in the high- vs. low-risk group (Figure 6E).

### Immune Checkpoint Gene Expressions and IC_50_ for Chemotherapy Agents

**FIGURE 7:**
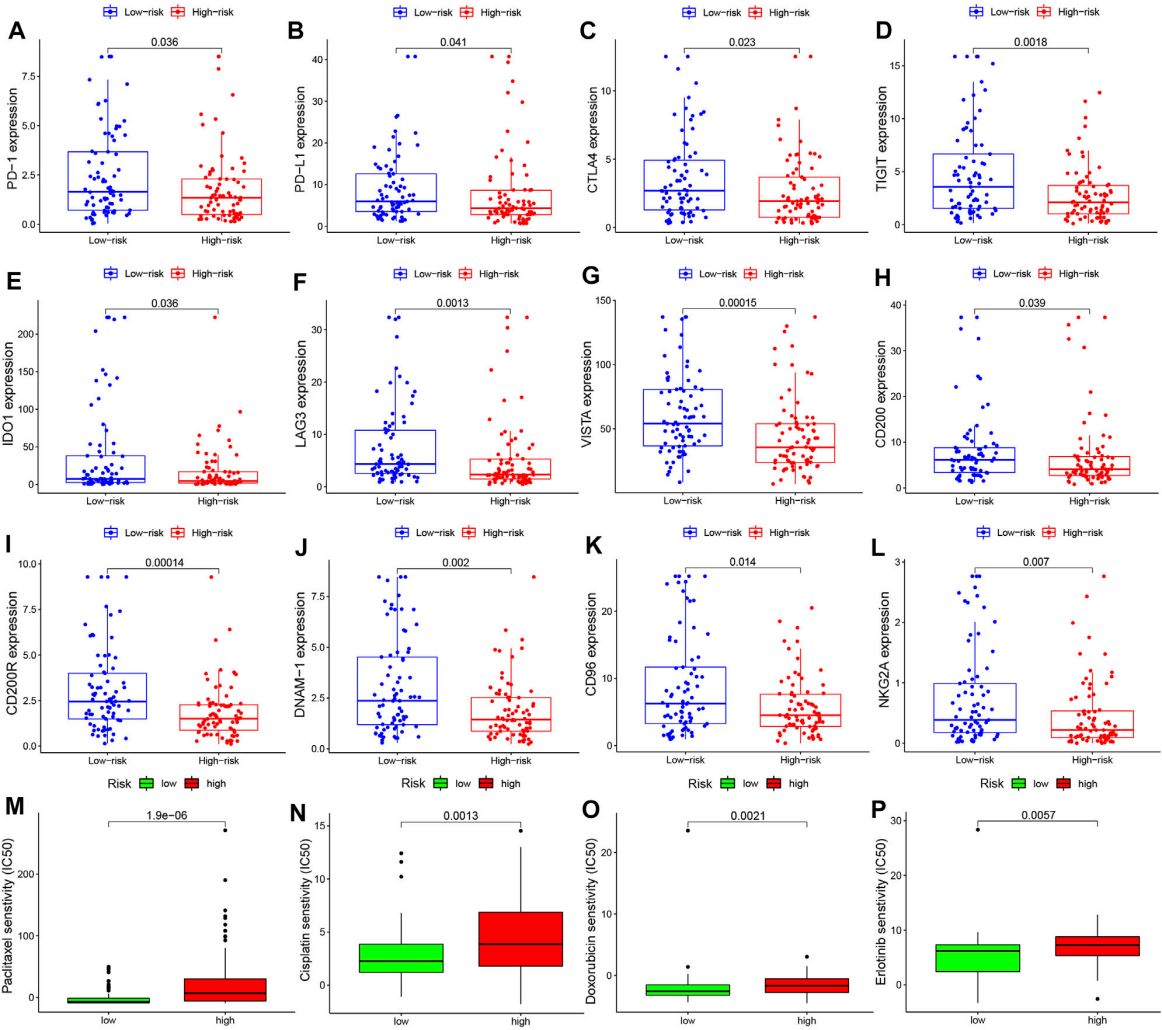
Immune checkpoint gene expressions and IC_50_ for chemotherapy and targeted therapy agents in EC. Expression levels of immune checkpoint genes **(A)** *PDCD1*, **(B)** *CD274*, **(C)** *CTLA4*, **(D)** *TIGIT*, **(E)** *IDO1*, **(F)** *LAG3*, **(G)** *VISTA*, **(H)** *CD200*, **(I)** *CD200R*, **(J)** *DNAM-1*, **(K)** *CD96*, and **(L)** *NKG2A*. Sensitivity to chemotherapy agents **(M)** paclitaxel, **(N)** cisplatin, and **(O)** doxorubicin and to the targeted therapy agent **(P)** erlotinib in high- and low-risk EC patients.

We compared expressions of 12 common immune checkpoint genes (*CD200*, *CD200R*, *CD274*, *CD96*, *CTLA4*, *DNAM-1*, *IDO1*, *LAG3*, *NKG2A, PDCD1*, *TIGIT*, and *VISTA*) between the two risk groups. All gene expressions were upregulated in the low- vs. high-risk groups (Figures 7A–L). The predicted IC_50_ for the three common chemotherapy agents (paclitaxel, cisplatin, and doxorubicin) and the targeted therapy agent erlotinib was also compared between the two groups. High-risk patients unanimously exhibited higher predicted IC_50_ for the four agents compared to low-risk patients (Figures 7M–P).

## Discussion

EC is considered most aggressive among gastrointestinal malignancies, which has a 5-year survival rate of 15–25% across the world [27, 28], with median overall survival of only 13 months [29]. EC patients may have cachexia, early satiety, dysphagia, aspiration, apastia, and other symptoms that pronouncedly diminish quality of life. Currently, immunotherapy comprised of immune checkpoint inhibitors, peptide vaccines, and adoptive T cell immunotherapy has received increasing attention [7], though it cannot substitute for conventional treatment like surgery, radiotherapy, and chemotherapy [30]. Its efficacy has been proven for some refractory EC subtypes [31]. For far too long, we have not yet identified an excellent biomarker for predicting immunotherapy response and OS, which emphasizes the need for future studies into exploring such biomarkers for EC. The prediction power of any single tumor marker is insufficient for tumors, whose microenvironment is jointly regulated by multiple genes. Although some studies have reported IRG signatures in EC [32,33], the current study performed extra analyses, including immune checkpoint biomarker expressions and IC_50_ prediction. Compared to previous reports, our study offers more feasible findings for clinical application, which can be directly used as recommendations for EC treatment. The seven-IRG-based prognostic signature exhibits good accuracy in predicting overall survival (with AUC ranging from 0.673 to 0.816), stages of cancer progression, and characterization of immune cell infiltration pattern. The signature also shows good performance in characterizing expression patterns of immune checkpoint genes and predicting IC_50_ values. The discrimination power allows physicians to select the optimal treatment strategies to hopefully extend the survival of EC patients.

The immune microenvironment is critical for tumor occurrence and progression. In the present study, we identified 275 DEIs based on gene expression data from 171 tissue samples of EC patients from TCGA. These genes are primarily involved in the activities of cytokines, chemokines, and growth factors, and their recognition and binding to protein targets, closely related to tumor growth, proliferation, or immunity. 15 of 275 prognostic DEIs were included in a Lasso-Cox model for optimal gene signature selection using 2/3 of all patients as a training cohort. A prognostic signature based on seven IRGs (*APLN*, *CACYBP*, *FABP3*, *GPER1, JAG2*, *SFTPA1*, and *XCR1*) was determined, and its efficacy in overall survival prediction was validated using the remanent 1/3 of patients assigned to the test cohort. Among others, the chemokine receptor XCR1 showed the highest specific gravity in the IRGs model (coef = −0.76193). It was reported that XCR1 was exclusively expressed on CD8^+^ DCs and can stimulate CD8^+^T cells to proliferate, thus enhancing the immune system [34]. XCR1 downregulation has been shown to stimulate the immune system to exert the antitumor activity, which is critical to alter the immune cell landscape in the TME that delays tumor progression [35]. It is expected to serve as a novel target of immunotherapy [36]. Calcyclin-binding protein (CACYBP) is universally considered most harmful, with its major function of participating in the connection between actin and tubulin to facilitate cytoskeleton formation [37] and in cell differentiation and proliferation in neuroblastoma NB2a cells via activation of the ERK1/2 pathway [38]. As highlighted in current studies, CACYBP promotes tumor occurrence and progression in various tumors via anti-apoptotic activity in cancer cells and inhibiting the cell cycle [39-41]. Fatty acid binding proteins 3 (FABP3) facilitates fatty acid transport, cell growth, cellular signaling, and gene transcription. Moreover, in non-small cell lung cancer and gastrointestinal stromal tumors, FABP3 overexpression is notably correlated with tumor size and lymph node metastasis of advanced tumors and with remarkably shorter survival of patients [42, 43]. It was therefore revealed that FABP3 is of strong prognostic significance. G protein-coupled estrogen receptor 1 (GPER1) has been reported to be involved in cell cycle regulation, endoplasmic reticulum stress, proliferation, apoptosis, and immune response [44]. *GPER1* silencing inhibits gastric cancer cell proliferation, migration, and invasion via inhibiting PI3K/Akt-mediated epithelial-mesenchymal transition [45]. A six-IRGs (*AMBP*, *C6*, *ITLN1*, *MADCAM1*, *PRLR*, and *TSPAN2*) based prognostic model in ESCC also obtained comparable predictive efficacy to our model [46]. The above genes are critical for EC progression, which can be considered to be oncogenes or tumor-suppressor genes. These gene studies may involve the key realm of EC development and management, but more studies are required for validation. The functions of genes mentioned above in tumor cells demonstrate that the signature is expected to predict the prognosis and progression stages of EC patients and hopefully offer opportunities to identify new therapeutic targets of EC to improve treatment. Overall, the signature is a biomarker associated with tumor immunity and growth inhibition.

We also assessed the prediction ability of the prognostic signature and ascertained its excellent accuracy in predicting the overall survival and death risk of EC patients and independent discrimination power from other clinical indicators. Further, it shows significant correlations with these clinical indicators. These findings suggest that the signature shows the potential of predicting how fast EC progresses based on especially clinical stages and lymph node and distant metastasis. High-risk patients need effective treatment in a more timely manner. Therefore, it can be involved in the early screening of high-risk EC patients for improved prognosis. Besides, in analyses of different pathological subtypes of EC, we found that EAC patients exhibited a higher risk score than ESCC patients, and the difference of the survival analysis was also found to be much more significant in the cases of EAC compared to ESCC. This finding indicates that the calculated risk score may be an important indicator in predicting the OS of EC cases, and the seven genes included in the signature can be important roles, probably in regulating the histological evolution of EAC, offering the prospect of identifying new targets for EAC treatment and prognosis prediction. Besides, its ability to predict somatic mutations has been assessed. The signature well predicted mutation probabilities between high- and low-risk EC patients, especially those of *TP53*, *TTN*, and *MUC16* genes. *TP53* mutations are most common in malignant tumors and associated with enhanced invasiveness and worse prognosis of patients [47, 48]. *TP53* mutations may stimulate tumor cell proliferation and tumor growth via abnormal p53/TGF-β signaling activity, potentially explaining rapid tumor progression and poor prognosis in high-risk patients. These predicted gene mutations may offer new targets for drug development or a new treatment strategy for patients at increased risk for tumor progression.

The seven-IRG-based signature also shows satisfactory efficacy in predicting ICG expressions, TMB, and the immune cell landscape in the TIME of EC patients, all of which are capable of sensitivity assessment of immunotherapy. Among the 12 ICGs frequently reported, PDCD1 (encoding PD-1) has been proven to facilitate EC progression and distant metastasis [11]. Patients with CD274 (encoding PD-L1) upregulation or high TMB tend to benefit more from immunotherapy therapy [12]. Although TMB has no effect on EC prognosis, it allows us to exclude the prognostic effect of TMB itself and turn our focus to the relationship between TMB and immunotherapy—the signature may somewhat compromise outcome in patients receiving immunotherapy. CTLA4- or IDO1-positive immune cells are negatively correlated with the overall survival of patients [49, 50]. Patients with VISTA-positive immune cells often exhibit prolonged survival [51]. However, those with CD274- and IDO1-double-positive immune cells have shorter overall survival and decreased sensitivity to chemotherapy agents [52]. The potential mechanisms of aberrant ICG expressions for tumor progression are complex and have not yet been fully explored. But PDCD1 and CD274 expressions as effective biomarkers for predicting ICIs’ efficacy have been ascertained by many [10-12,53]. In the present study, *CTLA4*, *CD274*, and *PDCD1* expressions were upregulated in low-risk patients. Neutrophils are most abundant in immune cells and a critical component of the TME. Neutrophil infiltration into tumors may allow inflammation to persist and fuel tumor progression and metastasis [54]. Mast cells promote tumor growth and proliferation via immunosuppression, angiogenesis, and tissue remodeling [55]. Consistently, our finding shows that activated mast cell and neutrophil infiltration was markedly enhanced in high- vs. low-risk patients, and naive B cell and resting mast cell infiltration was reduced. This indicates that the seven-IRG-based signature can characterize the TIME of high-risk EC patients. Numerous evidence shows that elevated PDCD1, CD274, and CTLA4 expressions are associated with a poor prognosis, which is in contrast to our findings of improved prognosis of low-risk patients who have the same gene expression patterns. This can be explained by the positive correlation of the infiltration of PDCD1-, CD274-, and CTLA4*-*overexpressed immune cells responsible for antitumor immunity with survival time. Studies have ascertained that PDCD1 and CD274 expressions in tumor cells and tumor-infiltrating immune cells are associated with improved prognosis, which supports our finding [56,57]. The above results indicate that the seven-IRG-based prognostic signature has good efficacy in predicting immune cell infiltration pattern, somatic mutations, TMB, and ICG expression pattern in EC. Taken together, it offers insight into the TIME of individual patients and molecular evidence to optimize ICI strategy for EC patients [58]. However, immune checkpoint expressions are not identical to the exact sensitivity to immunotherapy in patients, so its prediction for ICI efficacy still needs validation by future clinical trials.

In the present study, high-risk patients selected using the signature showed higher predicted IC_50_ for the chemotherapy agents paclitaxel, cisplatin, and doxorubicin and the targeted therapy agent erlotinib. Rather, low-risk patients had lower predicted IC_50_ for these agents. These results are consistent with a high frequency of *TP53* mutations described above as *TP53* has significant associations with the unsatisfactory efficacy of neoadjuvant therapy [59]. Therefore, we believe that this signature can offer helpful feedback to improve efficacy. Thus, the signature is promising in estimating the efficacy of conventional chemotherapy or targeted therapy strategies. Overall, low-risk patients identified by the signature hopefully benefit more from immunotherapy and conventional chemotherapy. As for high-risk patients, new therapeutic strategies or a combination of multiple agents are required to improve prognosis, which, however, calls for massive clinical observations for validation.

## Conclusion

The seven-IRG-based prognostic signature can independently predict the prognosis, tumor progression, and immune infiltration pattern in the TIME of EC patients. It shows the potential to monitor the efficacy of immunotherapy, chemotherapy, and targeted therapy for personalized treatment for these patients.

## Data Availability

The datasets presented in this study can be found in online repositories. The names of the repository/repositories and accession number(s) can be found in the article/Supplementary Material.

## Abbreviations

CACYBP, calcyclin-binding protein; CIBERSORT, Cell-type Identification By Estimating Relative Subsets Of RNA Transcripts; CTLA4, cytotoxic T-lymphocyte associated protein 4; DEGs, differentially expressed genes; EC, esophageal cancer; EAC, esophageal adenocarcinoma; ESCC, esophageal squamous cell carcinoma; FDR, the false discovery rate; GO, Gene Ontology; Half-inhibitory concentration, IC_50_; ICIs, immune checkpoint inhibitors; IRGs, immune-related genes; ICG, Immune checkpoint gene; IDO1, indoleamine 2,3-dioxygenase 1; KEGG, Kyoto Encyclopedia of Genes and Genomes; KM, Kaplan-Meier; LASSO, least absolute shrinkage and selection operator; NES, normalized enrichment score; OS, overall survival; PD-1, programmed death receptor 1; PD-L1, programmed cell death-ligand 1; TCGA, The Cancer Genome Atlas; TIME, tumor immune microenvironment; TME, tumor microenvironment; TMB, tumor mutation burden.
